# Molecular identification of *Mycobacterium avium* subspecies *paratuberculosis* in oral biopsies of Crohn’s disease patients

**DOI:** 10.1186/1757-4749-5-18

**Published:** 2013-07-10

**Authors:** Paola Molicotti, Antonio M Scanu, Aurea Lumbau, Sara Cannas, Alessandra Bua, Pietrina Lugliè, Stefania Zanetti

**Affiliations:** 1 Dipartimento di Scienze Biomediche – Microbiologia Sperimentale e Clinica, Università degli Studi di Sassari, Viale San Pietro 43/b, 07100 Sassari, Italy; 2 Dipartimento di Medicina Clinica Sperimentale ed Oncologica – Sezione Clinica Chirurgica, Università degli Studi di Sassari, Sassari, Italy; 3 Dipartimento di Scienze Chirurgiche, Microchirurgiche e Mediche. Università degli Studi di Sassari, Sassari, Italy

**Keywords:** MAP, Oral lesion Crohn, Oral granulomatous lesions, PCR IS*900*

## Abstract

Oral lesions may be found in patients with Crohn’s disease (CD), in a percentage up to 20%. The aim of this study was to investigate a possible relationship between *Mycobacterium avium* subsp. *paratuberculosis* (MAP) and oral lesions in CD patients. 23 oral biopsies were examined performing IS*900* Nested PCR; 9 of them were positive: 8 from CD patients and 1 from a control. Our purpose is to go on with this study, amplifying the number of subjects examined and testing subjects with oral lesions related to diseases other than CD to verify the specific association between MAP and oral lesions in CD patients.

## Background

**Figure 1:**
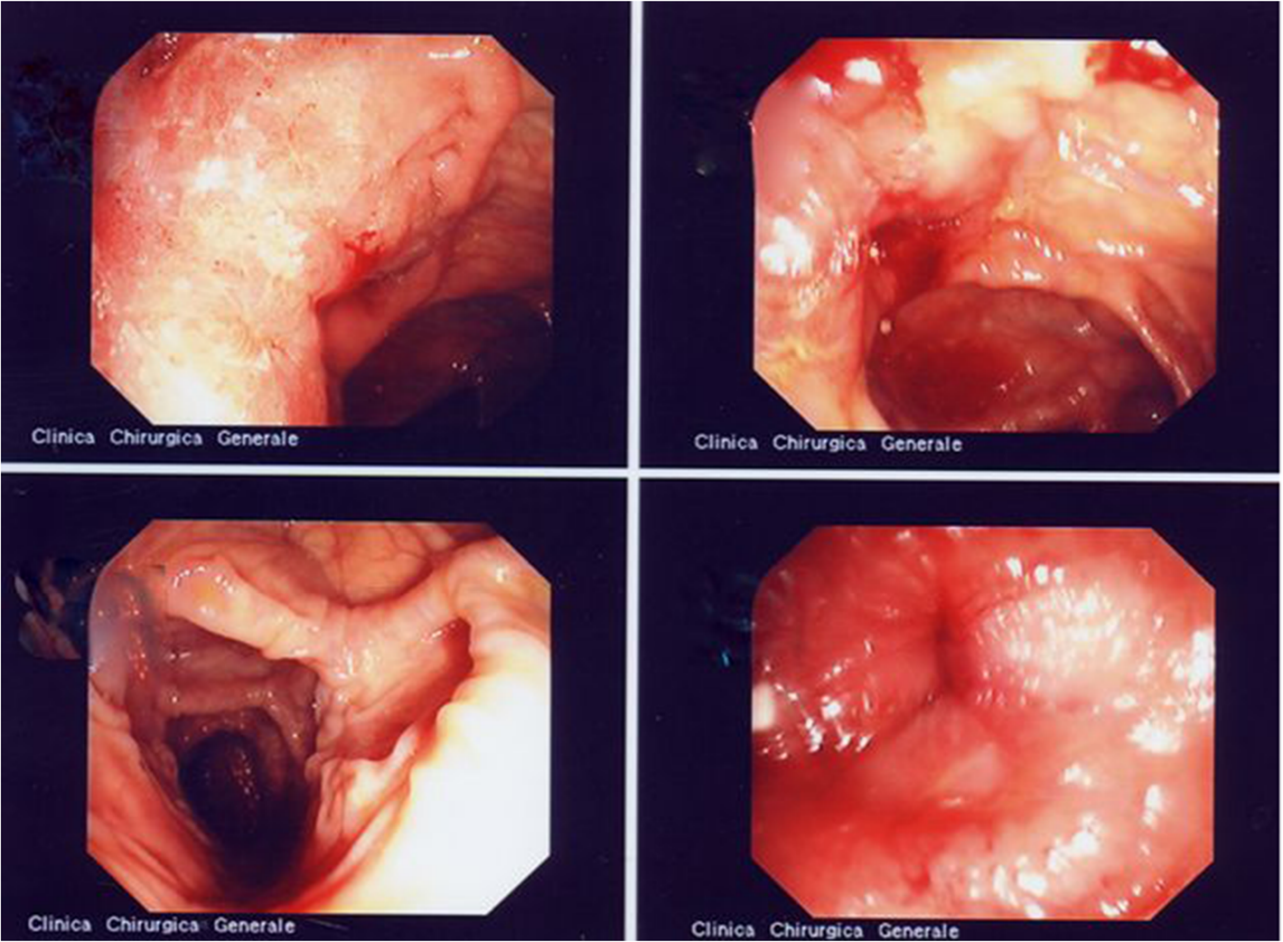
**Exulcerative lesions in ileal Crohn initially active with wall thickness, oedema with the bottom of afta covered with fibrin of the exulceration.** Istologic findings pathognomonic for CD.

**Figure 2:**
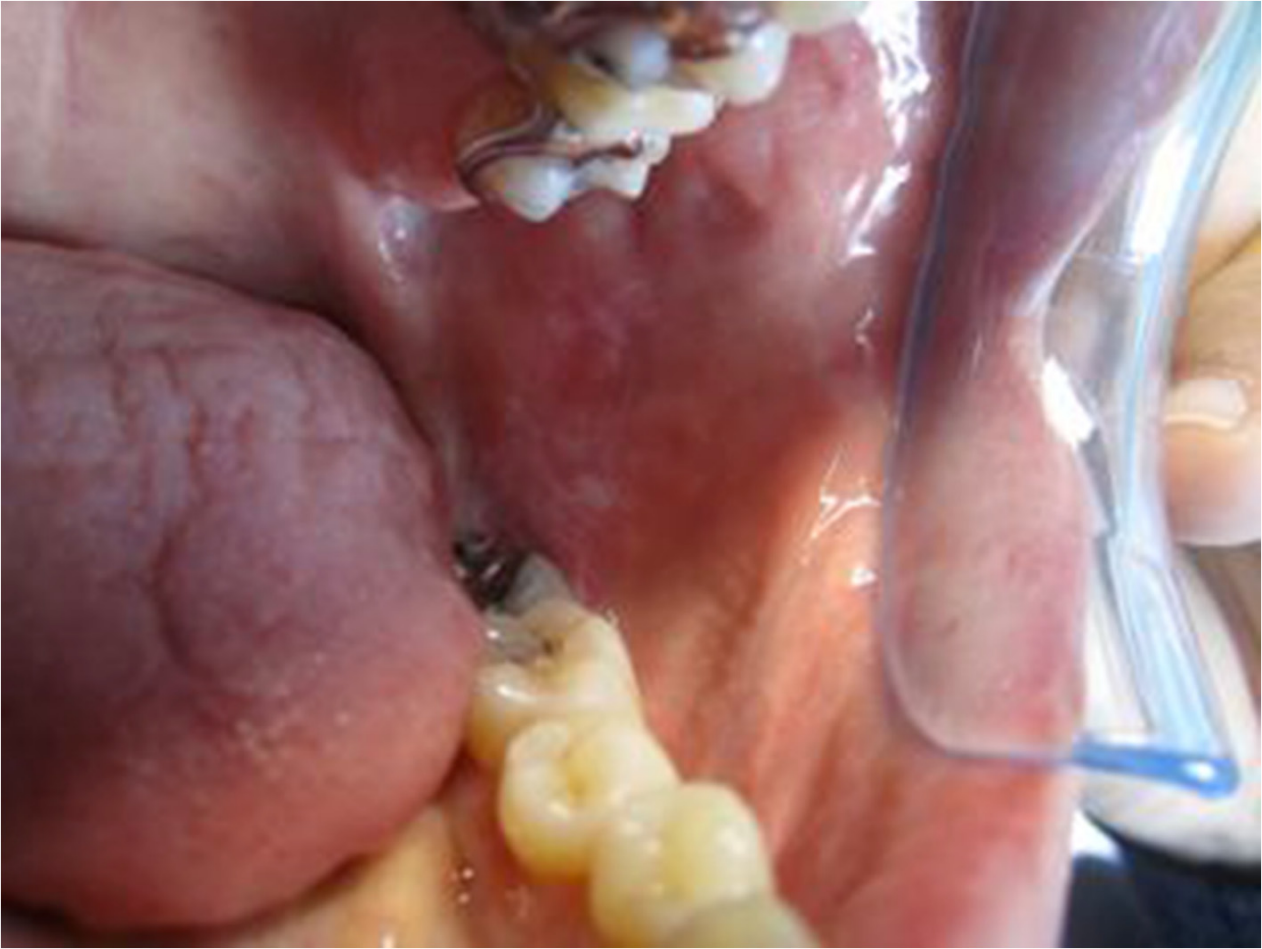
**Small aphthous exulcerative lesions in CD patients with wall thickness, oedema and fibrina deposit in the base of ulcer.** Istologic findings of CD.

Crohn’s disease (CD) is a chronic-recurring inflammatory bowel disease that may affect every part of the gastrointestinal tract. Oral lesions may be found in patients with full-blown intestinal CD or may represent the onset. It has been discussed about which lesions may be considered pathognomonic of CD or secondary nonspecific lesions: a histopathologic confirmation of the diagnosis is required (Figures 1 and 2). Several authors have hypothesized an involvement of *Mycobacterium avium* subspecies *paratuberculosis* (MAP) in CD in genetically predisposed subjects [1,2]. The aim of this study was to evaluate the presence of MAP in oral lesions of CD patients.

## Methods

23 Italian subjects were examined; a first group was composed of 12 CD patients, while a second one was made up of 11 healthy subjects. Subjects of both groups presented “aphthous type” oral lesions. Specimens were collected in physiological solution and sent to the Laboratory of Mycobacteriology of University of Sassari. The specimens were inoculated in liquid medium MGIT (Becton Dickinson), added with Mycobactin J at the concentration of 1μg/ml and in solid medium Herrold’s (Becton Dickinson). After DNA extraction following standard protocols, Nested PCR IS*900* was carried out (Table 1) [3]. Nested PCR was always performed with internal controls in order to avoid false positive results. Moreover, all the surfaces and the instruments used to perform PCR were cleaned every time, before and after their use with DNA Cleaner (DIATECH) to avoid cross-contamination. Nested PCR on biopsy samples was performed three times, and every time the results were the same. Statistical analysis was performed with Fisher’s exact test (Graph-pad Scientific calculator, p<0.05 was considered significant).

**Table 1:** **Results of Nested PCR IS*900***

**Patients**	**IS*900*Positive**		**IS*900*Negative**	
	**Women**	**Men**	**Women**	**Men**
CD	2	6	3	1
Control	0	1	4	6

## Results and discussion

**Figure 3:**
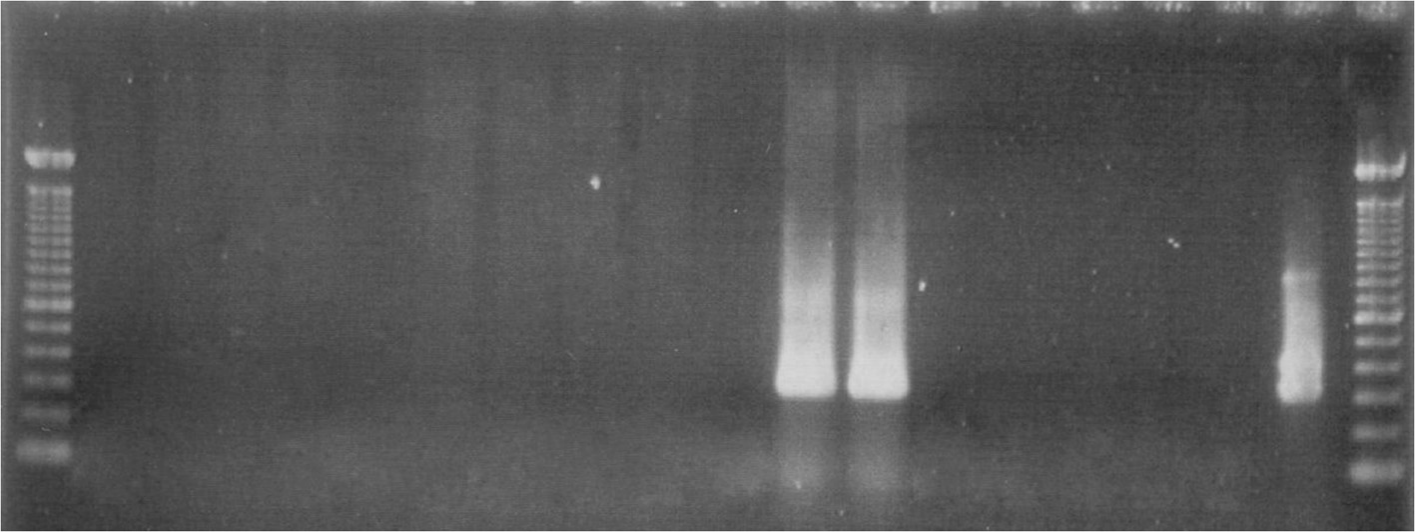
**Results of IS*900*PCR testing for *Mycobacterium avium *subsp. *paratuberculosis *in the patient CD with con oral lesions.** The results are consistent with an extensive *Mycobacterium avium* subsp. *paratuberculosis* infection. Lanes: 1 and 20 Markers 100 bp; Lane 2: negative control; Lane 19: positive PCR control; Lanes 12 and 13: positive samples.

The 12 patients affected by CD, 5 women and 7 men, aged 16–35 presented a full-blown ileal disease. 50% of patients (6/12) presented only an ileal location, 33% (4/12) showed a concomitant rectal location, while 17% (2/12), had also a duodenal location. In 67% of them (8/12) the oral lesions were compatible with an oral location of the disease, while in the remaining 33% (4/12) they were not. The subjects of the control group, 4 were women and 7 men, aged 45–65, had not any clinical sign of IBD or other gastroenteric disease. Cultural examination after 6 months was negative for all the biopsies. Nested PCR was positive in 9 biopsies: 8 from patients affected by CD and 1 from a subject of the control group (Figure 3 and Table 1). In the 8 CD patients the ulcerative lesions were compatible with oral CD. Histological examination was compatible with ulcerative lesions characterized by lymphoplasmacellular and histiocytarian chronic phlogosis. Neither cases of cobblestoning, nor cases in which swelling of lymph node chains were observed, while in 3 patients a mild lip swelling was observed. In 4 CD patients with a negative Nested PCR, oral ulcers were small secondary lesions characterized by a little flat exulceration with hyperaemic edge and intensely painful. Histological examination was compatible with small abscessual foci or nonspecific phlogosis with chorion oedema and neutrophilic cells infiltration.

The subjects of the control group presented an aphthous-like lesion with hyperaemic edge and a bottom covered with fibrin, without a histological picture compatible with CD. In the remaining 10 control subjects with a negative Nested PCR, lesions varied from simple gingivites to aphthous gingival-stomatites or to small aphtha-like exulcerative lesions; in no case the lesions showed a CD-like histology. Statistical analysis showed a significative difference between the positive results of Nested PCR obtained in the first group and in the control one (p=0,0094 two tailed). The molecular methods specific for MAP allow its identification from intestinal biopsies of CD patients and represent the gold standard to distinguish MAP from other mycobacteria [4-7]. Oral lesions in CD patients occur in a percentage comprised between 0.5% and 20% [8-10] and it has been seen that some aphthous-like lesions may be specific indexes of the disease [11,12]. The cultures resulted negative probably due to difficulties in the isolation of MAP. The negative results of the cultural examination may be explained with the difficulties in the isolation of MAP known in literature. Its growth is extremely slow (4–6 months) and strongly dependent on the addition of Mycobactin J in the culture medium. Instead, Nested PCR was positive in 8 of 12 CD patients and in 1 healthy subject. Positiveness associated to the control subject requires further investigation to ascertain if the oral lesion is an initial sign of the disease. We found a highly significant association between *Mycobacterium avium* subsp. *paratuberculosis* and oral biopsies in CD patients, and considering that these lesions may occur at the beginning of CD and that MAP could be the etiological agent, its quick identification might represent an important diagnostic tool.

## Competing interests

The authors declare that they have no competing interest.

## Authors’ contributions

PM was involved in the experimental design and drafted the manuscript. AS was involved in the experimental design and helped to draft the manuscript. AL participated in the design of the study. SC carried out the samples’ analysis. AB provided writing assistance. PL and SZ conceived the study, and participated in its design and coordination and helped to draft the manuscript. All authors read and approved the final manuscript.
